# Androgen Receptor and PI3K Pathway Activity in Ovarian Cancer

**DOI:** 10.17303/jcrto.2019.7.103

**Published:** 2019-05-07

**Authors:** Addie Hill, Mihaela Cristea, Miaoling He, Paul Frankel, Susan Neuhausen, Sumanta K Pal, Jeremy O Jones

**Affiliations:** 1 Department of Medical Oncology & Therapeutics Research, City of Hope Comprehensive Cancer Center, Duarte California; 2 Division of Molecular Pharmacology, Department of Medical Oncology & Therapeutics Research, City of Hope Comprehensive Cancer Center, Duarte California; 3 Division of Biostatistics, Department of Computational and Quantitative Medicine, City of Hope Comprehensive Cancer Center, Duarte California; 4 Department of Population Sciences, Beckman Research Institute, City of Hope Comprehensive Cancer Center, Duarte California

## Abstract

We sought to evaluate androgen receptor (AR) and PI3K pathway activity in ovarian cancer cell lines and tissue and determine if either pathway was correlated with growth of ovarian cancers. AR expression and activity were quantified using immunohistochemistry (IHC) and RT-qPCR in six ovarian cancer cell lines and 51 tissue samples. Phospho-mTOR and AKT expression were quantified by IHC as well. Cell growth was assessed in the presence of AR modulating drugs and metformin. We found that despite robust AR expression and activity, no cell line was dependent on androgen for growth. However, metformin inhibited activity in five of the six cell lines. Patient tissues had large variation in AR expression and activity, as well as in expression of phospho-mTOR and AKT, but none of these variables correlated with progression-free survival (PFS). AR expression and activity did not predict the dependence of ovarian cancer cell lines on androgens for growth, and AR expression and activity did not correlate with PFS. This result suggests that AR expression as a criterion for patient selection for clinical trials evaluating molecules targeting AR may not predict response for ovarian cancer patients.

## Introduction

Epithelial ovarian cancer (EOC) is the most lethal gynecologic malignancy with 22,530 new cases and 13,980 deaths expected in 2019 [1]. Fewer than 25% of cases are diagnosed in the early stages. Treatment for ovarian cancer involves surgical de-bulking, followed by platinum/taxane-based chemotherapy. Although most patients initially respond to platinum-based chemotherapy, the majority of patients relapse and eventually develop drug-resistant disease. Estrogen and progesterone receptors (ER and PR) are widely expressed in normal and tumor ovarian tissues [2,3] For this reason, tamoxifen and aromatase inhibitors have been utilized in patients with recurrent ovarian cancer who have progressed after platinum-based chemotherapy [4]. These agents are administered to patients with isolated biochemical relapse [5] or evidence of tumor progression [6]. However, ER/PR expression does not always correlate with response to hormonal therapy [7]. Interestingly, the androgen receptor (AR) is more widely expressed in ovarian cancer than either ER or PR, [2,3,8] in up to 85% of cancers. While AR expression was not associated with better survival when all histological subtypes of cancer were considered, [2,9] AR expression was associated with better disease-specific survival in the serous subtype, regardless of the differentiation grade [9]. Such findings led to a study (NCT01974765) evaluating single agent enzalutamide, an AR antagonist, in AR-positive ovarian cancers (defined as ≥5 % positivity by IHC), regardless of histological subtype.

Several observational studies have reported improved survival in cancer patients taking metformin in several malignancies including ovarian cancer [10]. The anti-proliferative effect of metformin may be, at least, partially due to the inhibition of the PI3K/AKT/mTOR signal transduction pathway [10]. Interestingly, it has been shown quite convincingly in prostate cancer models that AR and PI3K signaling inhibit each other in a reciprocal fashion, and when one pathway is inhibited, the other becomes more active [11]. There are several clinical trials in prostate cancer currently assessing the benefit of targeting the AR and PI3K/AKT pathways simultaneously. It is possible that the same reciprocal relationship exists in ovarian cancer and that a similar combination treatment with an AR inhibitor, enzalutamide and a PI3K/AKT pathway inhibitor, metformin, could benefit ovarian cancer patients.

In this study, we quantified the expression and activity of AR and PI3K/AKT pathways in ovarian cancer cell lines and tumor tissue samples and examined the sensitivity of the cell lines to enzalutamide and metformin. Our findings have important implications for ongoing trials with these agents.

## Materials and Methods

### Cell culture:

Cells were provided by collaborators but were originally obtained from the ATCC. SKOV3 cells were maintained in McCoy's 5a Medium Modified with 10% FBS. A2780 cells were maintained in RPMI-1640 with 10% FBS. OV-90 cells were maintained in a 1:1 mixture of MCDB 105 medium containing a final concentration of 1.5 g/L sodium bicarbonate and Medium 199 containing a final concentration of 2.2 g/L sodium bicarbonate, with 15% FBS. OVCAR3 cells were maintained in RPMI-1640 supplemented with 20% FBS and 0.01mg/ml insulin. OVCAR8 cells were maintained in DMEM with 10% FBS. COV362.4 cells were maintained in DMEM with L-glutamine (300mg/L) and 10% heat inactivated fetal bovine serum. For some studies, cells were transferred to media containing charcoal dextran treated (C/S) FBS. Luciferase assays: Cells were transfected using Lipofectamine LTX & Plus (Thermofisher) with PSA-luciferase [12] and pRL-SV40 (Promega) plasmid as a control. Cells were transferred to quadruplicate wells of a 96-well plate in C/S medium 24 hours after transfection and treated with 1 nM DHT, 1 uM enzalutamide, or 10 mM metformin. Luciferase activity was assayed 24 hours after treatment using the dual-luciferase reporter assay system (Promega) on a Tecan fluorescent plate reader. ANOVA methods with a Tukey’s correction for planned comparisons were used to determine significant differences between treatment groups.

### RT-qPCR and AR activity score:

RNA was isolated from cultured cells or from cancerous and benign tissue sections, as marked by the study pathologist, of an unbaked FFPE slide using the FFPR RNA easy kit (Qiagen), as we have done previously [13]. RNA was reverse transcribed and relative target-gene expression was assessed by quantitative-PCR (qPCR) with a SYBR green detection dye (Invitrogen) and Rox reference dye (Invitrogen) on the Step One Real Time PCR System (Applied Biosystems). Using the ΔΔCt relative quantification method, target gene readouts were normalized to RPL19 and GADPH transcript levels. Experiments were the average of biological triplicates. Target genes included *AR* and a 20-gene panel that has been previously used to define an “AR activity score;” [14] this 20 gene panel itself was derived from previously published AR activity gene panels [15,16]. We found that 12 of these 20 genes (*FKBP5, MED28, ELL2, KLK2, PMEPA1, CENPN, C1ORF116, NKX3.1, KLK3, EAF2, TMPRSS2, HERC3*) were robustly expressed and androgen-induced in the six ovarian cancer cell lines. The transcript levels, relative to housekeeping genes, were compared to those from LNCaP cells, a prototypical prostate cancer cell line with robust AR activity, and the mean percent of LNCaP transcript levels was reported as the AR activity score.

### Cell proliferation assays:

For growth curves, cells were transferred to C/S medium three days before they were divided and plated at a density of approximately 20,000 cells/well in 48 well plates, in quadruplicate. The following day, vehicle or drugs were added to the cells. Proliferation was determined by measuring the DNA content of the cells in each well. Cells were fixed in 2% PFA, followed by staining for 5min at RT with 0.2ng/mL 4',6-diamidino-2-phenylindole (DAPI) in PBS. The cells were washed with PBS, then read on a fluorescence plate reader (FPR) using 365/439 excitation/emission wavelengths. A Student’s t test was used to determine significant differences among populations.

### Western blot assay:

Cell lysates were resolved via SDS-PAGE and transferred to nitrocellulose membranes. Membranes were blocked in 5% milk and probed overnight with antibodies including AR (PG21, Millipore), phospho-Ser473 AKT (GeneTex), phospho-Ser2448 mTOR (GeneTex), or controls: p84, actin, or GAPDH (GeneTex).

### Patient materials and IHC:

Patient slides and clinical data were collected under City of Hope IRB16430. IHC was performed using the same antibodies as used for Western blotting. The “IHC score” was determined by taking the average percentage of positively staining cells x the staining intensity (0-3) across eight 40x-fields within the marked cancerous area. Example images were obtained using an Aperio Digital Pathology Slide Scanner.

### Statistical analysis:

Data were analyzed using a Student’s t test. All analyses were performed at a significance level of *P<0.05. Experimental data are presented as the mean plus or minus standard error.

## Results

### Cultured ovarian cancer cell lines are not dependent on androgens for growth, despite AR expression and activity

**Figure. 1:**
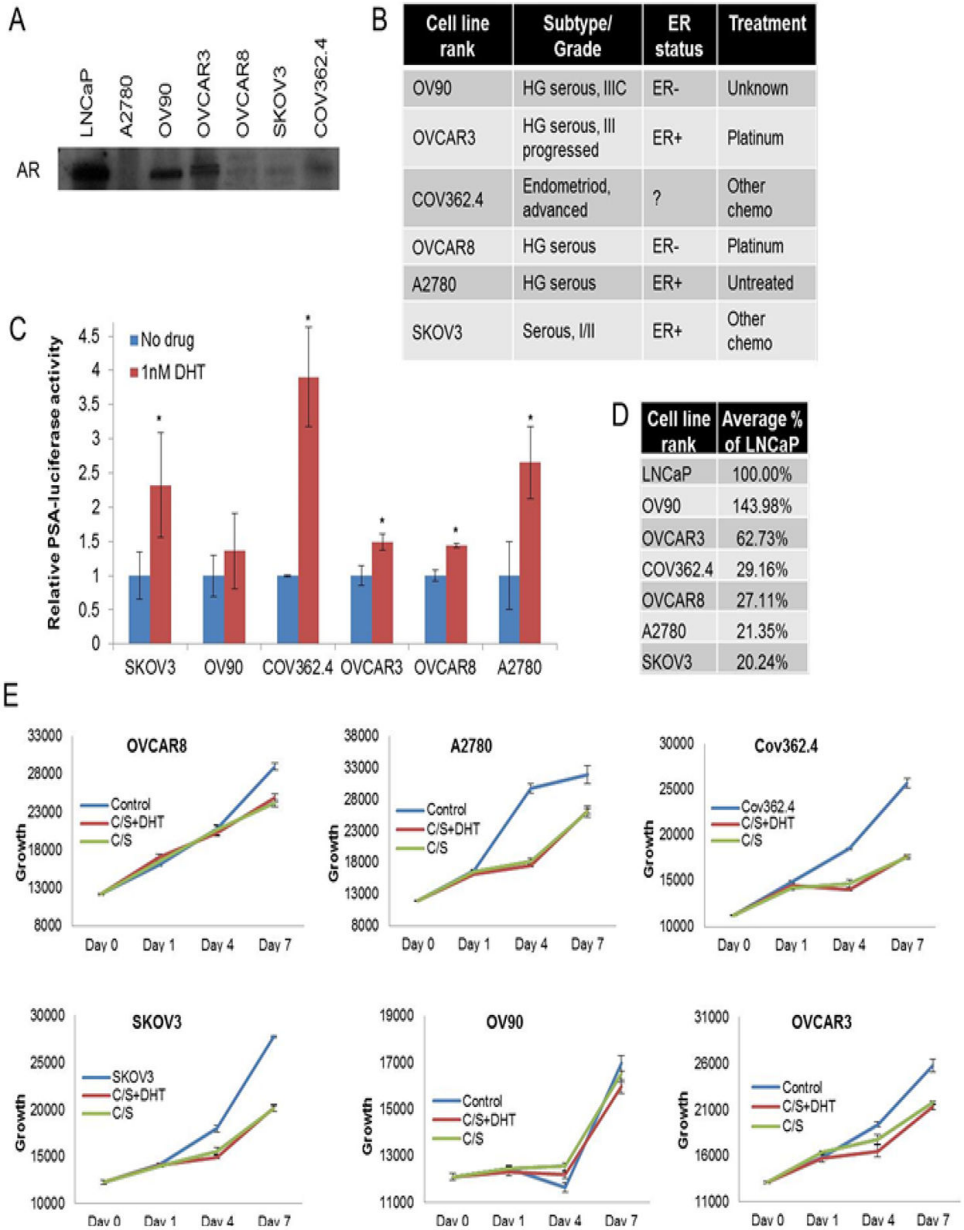
A) Western blot for AR expression. (B) Cell line information. (C) Cells were transfected with the androgen-responsive and control luciferase plasmids, treated with vehicle or DHT, and luciferase activity was quantified (* p<.05). (D) AR activity score of each cell line in comparison to LNCaP cells. (E) Cell growth was quantified following the indicated treatments *(C/S charcoal stripped FBS).

The AR is widely expressed in the normal ovarian epithelium [17] and also has been reported to be expressed to varying degrees in ovarian cancer epithelial cells [2,3,8]. Many cell lines derived from cancers express AR as well [18]. We used a panel of AR-expressing ovarian cancer cell lines to test whether AR expression and activity lead to androgen-dependent growth of these cells. AR was expressed at varying levels (Figure. 1A) across cell lines derived from tumors with different histologies and from patients with different treatment histories (Figure. 1B). Using a luciferase expression plasmid driven by an androgen-responsive promoter [12], we examined the activity of AR in these cell lines in response to the potent AR agonist, dihydrotestosterone (DHT) (Figure. 1C). All cell lines had DHT-induced activity to varying degrees. As this read-out represents only one promoter, we also adapted the “AR activity score” [14] for use in the ovarian cancer cell lines. We measured the levels of 12 androgen-responsive transcripts, normalized to two house-keeping genes, in each of the cell lines and compared them to the levels of those same transcripts in the canonical androgen-responsive LNCaP prostate cancer cell line (Figure. 1D). We found that the ranking of cell lines by AR activity score was very similar to the ranking of cell lines by level of AR expression. We next examined the androgen-dependent nature of the growth of the cell lines by culturing them in media containing full serum, media containing charcoal/dextran treated serum to remove hormones, and media containing the treated serum plus DHT to induce AR activity (Figure. 1E). Interestingly, the treated serum reduced the growth of all cell lines, but addition of DHT did not restore the growth. These data strongly suggest that despite high levels of AR expression and activity, growth of these cells is not dependent on androgens. The fact that the treated serum reduced growth suggests that growth is dependent on other growth factors that were removed along-side androgens with the charcoal/dextran treatment.

**Figure. 2:**
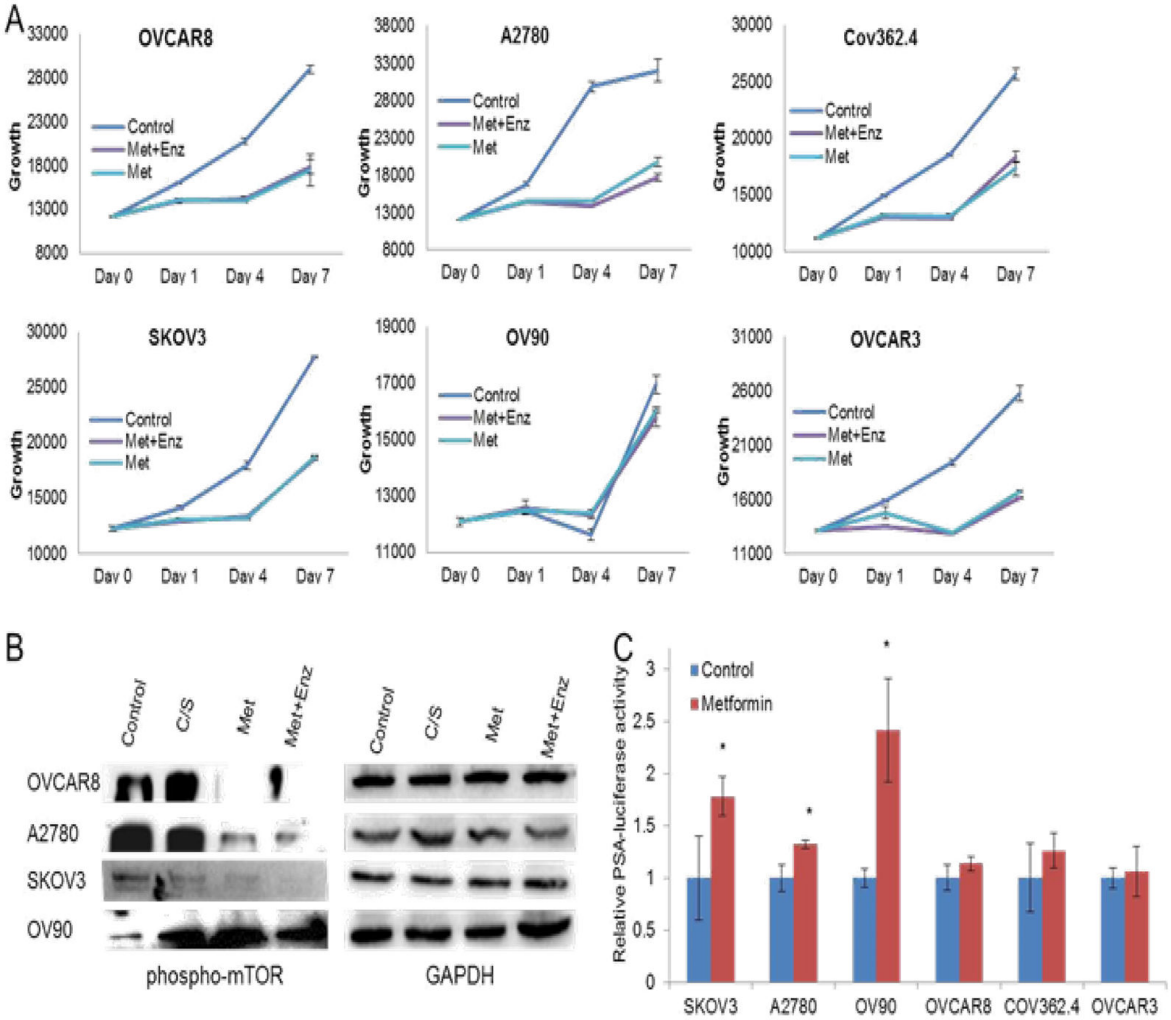
(A) Cell growth was quantified following the indicated treatments *(met= 10mM metformin, enz= 1uM enzalutamide). (B) Western blot for indicated proteins from cell lines treated with C/S serum, met, enz, or the combination. (C) Cells were transfected with the androgen-responsive and control luciferase plasmids, treated with vehicle or metformin, and luciferase activity was quantified (* p<.05).

As metformin has shown activity in ovarian cancer patients, growth in the panel of ovarian cancer cell lines was examined in the presence of this drug. All cell lines examined, except OV90, were extremely sensitive to metformin (Figure. 2A). The OV90 cell line contains a BRAF mutation that causes high constitutive PI3K and mTOR activity,[19] which may allow it to bypass the effects of metformin, although it was still slightly inhibited by the drug. Western blot analysis demonstrated that metformin decreased the levels of phospho-mTOR, a key downstream marker of PI3K activation, [20] in three sensitive cell lines, but not OV90 (Figure. 2B).

There is a well-documented inverse relationship between AR and PI3K activity in prostate cancer; when AR is inhibited, it increases PI3K activity and vice-versa [11]. To investigate if such an interaction exists in ovarian cancers, we first treated ovarian cancer cells with metformin and examined the effects on AR activity. Metformin treatment increased AR activity to varying degrees in all cell lines (Figure. 2C), suggesting that PI3K inhibition increases AR activity. However, addition of enzalutamide, the AR inhibitor, to the ovarian cancer cells did not increase levels of phospho-mTOR (Figure. 2B). Concurrent inhibition of PI3K and AR in prostate cancer models has been shown to improve responses compared to either single agent alone [11]. However, addition of enzalutamide to metformin did not improve the ability of metformin to inhibit growth of the ovarian cancer cell lines (Figure. 2A), suggesting again that these cells are not dependent on AR for growth and that a reciprocal interaction between PI3K and AR signaling does not occur in ovarian cancer cells.

### AR Expression and AR Activity Are Not Correlated in Human Tissue and Neither Correlates with PFS In High Grade Serous Ovarian Cancer

**Figure. 3:**
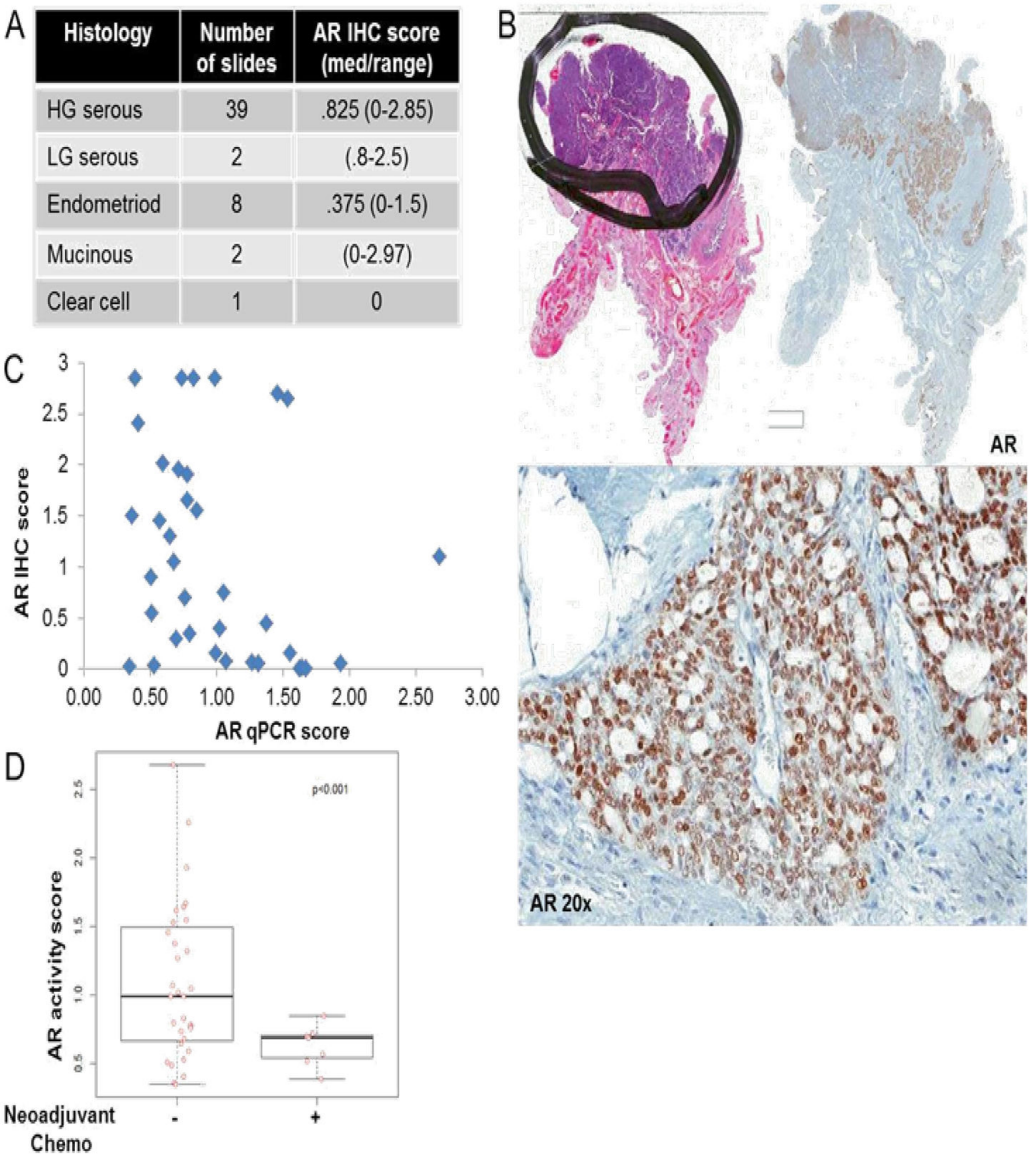
(A) IHC median score and range for each histological subtype. (B) Example whole slide H&E and AR IHC staining (top) with 20x magnification AR staining (bottom) (C) AR IHC score plotted against the AR activity score for HG serous samples (D) Plot of AR activity score in samples with or without neoadjuvant chemotherapy exposure.

We performed IHC for AR in 51 primary ovarian cancer tissue samples representing 48 patients (some with mixed histologies), including low and high grade serous, endometroid, clear cell, and mucinous subtypes, the majority of which were high grade serous (Figure. 3A). Using an intensity multiplied by area scoring system, we found a wide range of AR expression in the ovarian cancers (Figure. 3A, B). We then isolated >90% pure cancerous tissues from the high-grade serous samples and prepared RNA for RT-qPCR of AR-regulated genes. We found a range of AR activity in the samples, based on the AR activity scores (median = 0.81, range 0.35-2.68). Interestingly, there was not a strong correlation between AR staining by IHC and the AR activity score (Figure. 3C).

We also performed IHC for phospho-mTOR and phospho-AKT, another key downstream marker of PI3K activation, on slides of high-grade serous ovarian cancer tissue and scored them using the same system as for AR IHC. Again, we found a wide range of expression among the high-grade serous samples for both phospho-mTOR (median = 1.65, range = 0.2-2.7) and phospho-AKT (median = 1.35, range = 0.1-2.3). To determine if an inverse relationship existed between the PI3K and AR pathways in the high-grade serous cancer tissues, we looked for correlations between AR IHC or AR activity scores and IHC scores for the phosphoproteins. There were no significant correlations. We next investigated the relationship between the IHC and qPCR scores and clinical variables in 38 high-grade serous patients. None of our markers correlated with PFS. However, the AR activity score was lower tumors of patients assessed after neoadjuvant therapy (n=7) than in tumors of patients assessed prior to receiving any chemotherapy (n=31) (mean 0.63 vs 1.05, p<0.001, t-test) (Figure 3D). This suggests decreased AR activity after exposure to chemotherapy.

Furthermore, we found that AR expression was associated with BRCA1 and BRCA2 status. The mean AR expression in patients with peathogenic BRCA mutations (n=6) was higher compared to the mean AR expression in non-BRCA mutation patients (n=29) (1.27 vs 0.14, p<0.001, t-test).

## Discussion

The AR is known to be expressed in ovarian cancer cells, but little is known about the impact of AR expression and activity on the natural history of the disease. In this study, AR expression and degrees of increased signaling activity in response to an androgen agonist, DHT, varied among the six ovarian cancer cell lines. A more comprehensive assessment of AR signaling, the AR activity score, also was evaluated and correlated with AR expression. However, hormonal manipulation of the cell lines suggested that AR stimulation alone was not sufficient to promote growth of these cell lines.

We also evaluated whether AR expression or AR activity of their tissue correlated with PFS in 38 patients. Neither AR expression nor the AR activity score was correlated with PFS. The patient samples examined were high-grade serous adenocarcinoma histology, which limits the application of our findings to other ovarian cancer histologic subtypes. Unlike the cell lines, AR expression did not correlate strongly with the AR activity score in tissue samples. This may simply reflect differences in the techniques, or it may imply the existence of cancers with incongruous AR expression and activity, analogous to “AR indifferent” prostate cancers that arise more frequently in highly-treated patients [21].

It is possible that the genes used to define the AR activity score are less relevant in ovarian cancer than they are in prostate cancer. One or several of these genes may be more strongly regulated by transcription factors other than AR in ovarian cancer. This may explain why several samples that had little AR expression detected by IHC still had high AR activity scores. Our results from the cell line experiments suggest caution should be used when using AR expression or activity as eligibility criteria for patient selection on clinical trials that are evaluating molecules targeting AR in ovarian cancer. AR expression or activity may not reflect cancer cell dependency on the androgen pathway and therefore may not predict response to AR antagonists or AR modulators.

In breast cancer, AR expression is associated with improved overall survival regardless of subtype of breast cancer or co-expression of ER. In triple negative breast cancer (TNBC), AR expression is seen in 12-55% of cases and is being evaluated as a therapeutic target in this patient population; in a recent phase II trial of TNBC with AR expression, there was a clinical benefit rate of 35% at 16 weeks and a median PFS of 14.7 weeks with enzalutamide [22]. The result in TNBC is different than our results in ovarian cancer. Our results suggest that AR expression did not impact PFS in patients with high-grade serous ovarian cancer and therefore, does not appear to be an independent prognostic factor. However, similar to studies in TNBC, we found that average AR expression was higher in *BRCA1* and *BRCA2*-mutated high-grade serous ovarian tumors compared to non-mutated *BRCA* tumors. This is consistent with published data suggesting that *BRCA1* status correlated with AR expression in TNBC. Decreased expression of AR has been measured in *BRCA1*-wild type TNBC cells [23] and suggests that *BRCA1*-mutated malignant cells may have up-regulated AR.

Other evaluations using the ovarian cancer cell lines included assessing their sensitivity to metformin. All cell lines were sensitive to inhibition by metformin, except for OV90. The OV90 cell line has a BRAF mutation that causes increased PI3K activity, effectively bypassing metformin. These results are consistent with a growing body of literature on the anti-tumor effects of metformin. Metformin has been shown to inhibit ovarian cancer growth and increases sensitivity to paclitaxel in mouse models [24]. In 341 ovarian cancer patients, those with diabetes treated with metformin had longer PFS [25]. An ongoing randomized phase II trial is evaluating the addition of metformin in conjunction with standard carboplatin plus taxol followed by metformin maintenance in ovarian cancer (NCT02122185). The beneficial effects of metformin extend to other tumors as well. In a colorectal cancer patient population, patients with diabetes treated with metformin had superior overall survival [26].

Finally, we investigated the interaction between the PI3K/AKT and AR pathways in ovarian cancer cell lines treated with metformin and enzalutamide. Increased activation of the PI3K/AKT pathway conferred cisplatin resistance in an ovarian cancer cell line,[27] implicating the PI3K/AKT pathway in ovarian cancer cell survival and evasion of apoptosis. In 2011, Carver at el studied the cross-talk between AR and PI3K/AKT pathways in PTEN-deficient prostate cancer. They found reciprocal feedback regulation of PI3K and AR signaling whereby AR inhibition activated AKT signaling by reducing levels of the AKT phosphatases and PI3K pathway inhibition activated AR signaling by reducing feedback inhibition of HER kinases. Furthermore, simultaneously inhibiting both the AR and PI3K/AKT pathways caused dramatic reductions in tumor volume in prostate cancer models, including PTEN-deficient mice and human xenografts [11]. We hypothesized that this reciprocal relationship between the AR and PI3K/AKT pathways would exist in ovarian cancer and if present, may respond to treatment with dual suppression.

We evaluated 6 ovarian cancer cell lines and 51 tumor samples from patients with ovarian cancer of various histologies, predominantly high-grade serous adenocarcinoma which comprises more than 70% of ovarian cancers. Although the AR activity score increased to varying degrees in all cell lines exposed to metformin, the activity of the PI3K pathway, as measured by phospho-mTOR, was not increased by the addition of enzalutamide, an AR antagonist. Although there appeared to be some interaction between AR and PI3K signaling in the ovarian cancer cell lines, it was not reciprocal as it is in prostate cancer. Assessment of additional markers of AR and PI3K signaling, and use of PI3K inhibitors that are more selective than metformin, would be useful to clarify the relationship between these pathways in ovarian cancer.

The limitations of our study include the small sample size and limited representation of other histologic subtypes except for high-grade serous adenocarcinoma in patient samples. While our study did not support robust reciprocal feedback between AR and PI3K/AKT pathway activity in ovarian cancer, the small sample may prevent this finding from being extrapolated and further studies are warranted in different ovarian cancer histologies. Other limitations include some inherent subjectivity in quantifying immunohistochemistry and technical difficulties in performing and interpreting phospho-proteins by IHC in FFPE tissue.

In summary, in our evaluation of 6 ovarian cancer cell lines and 51 ovarian cancer patient tissue samples, AR expression and AR activity did not consistently correlate with each other, growth of cancer cells, or PFS. Caution must be exercised when using AR expression as a selection criterion for clinical trial participation as a way to predict response to AR antagonists. Our study corroborates growing literature supporting metformin as an anti-tumor agent in ovarian cancer. A reciprocal relationship between the AR and PI3K pathway in ovarian cancer is not yet confirmed as it is in prostate cancer.
